# Characteristics of Sodium Alginate/Antarctic Krill Protein Composite Fiber Based on Cellulose Nanocrystals Modification: Rheology, Hydrogen Bond, Crystallization, Strength, and Water-Resistance

**DOI:** 10.3390/gels8030139

**Published:** 2022-02-22

**Authors:** Jicheng Shan, Jing Guo, Fucheng Guan, Feng Li, Chunqiu Di

**Affiliations:** 1 School of Textile and Material Engineering, Dalian Polytechnic University, Dalian 116034, China; sjc961122@163.com (J.S.); 15621588215@163.com (F.L.); dcq89757@163.com (C.D.); 2 Liaoning Engineering Technology Research Center of Function Fiber and Its Composites, Dalian Polytechnic University, Dalian 116034, China

**Keywords:** cellulose nanocrystals, sodium alginate, antarctic krill protein, structural viscosity index, wet spinning

## Abstract

The purpose of adding cellulose nanocrystals (CNCs) into sodium alginate (SA) and Antarctic krill protein (AKP) system is to use the ionic cross-linking of SA and AKP and the dynamic hydrogen-bonding between them and CNCs to construct multiple cross-linking structures, to improve the water-resistance and strength of SA/AKP/CNCs composite fiber. Based on the structural viscosity index in rheological theory, the ratio of spinning solution and temperature were optimized by studying the structural viscosity index of the solution under different CNCs content and temperature, then the composite fiber was prepared by wet spinning. We found that when the content of CNCs is 0.8% and 1.2%, the temperature is 45 °C and 55 °C, the structural viscosity is relatively low. Under the optimal conditions, the intermolecular hydrogen bonds decrease with the increase of temperature. Some of the reduced hydrogen bonds convert into intramolecular hydrogen bonds. Some of them exist as free hydroxyl; increasing CNCs content increases intermolecular hydrogen bonds. With the increase of temperature, the crystallinity of composite fiber increases. The maximum crystallinity reaches 27%; the CNCs content increases from 0.8% to 1.2%, the breaking strength of composite fiber increases by 31%. The water resistance of composite fiber improves obviously, while the swelling rate is only 14%.

## 1. Introduction

Cellulose is one of the most abundant natural polymers globally that can be chemically treated to produce cellulose nanocrystals (CNCs) [1]. CNCs have the excellent properties of cellulose and have the advantages of nanomaterials such as high strength, high modulus, and large specific surface area [2]. There are a large number of active hydroxyl groups on the surface of CNCs, which can participate in the formation of hydrogen bonds and various chemical cross-linking; therefore, CNCs has attracted increasing attention in the field of nanocomposites [3], and it’s considered as the best filler reinforcement material in the 21st century [4]. Ye et al. [5], added CNCs to poly(butylene succinate) (PBA) and found that CNCs could promote the crystallization and lamellar thickening of PBA. This mechanism attributes to the “memory effect” of CNCs/PBA composite in the molten state, namely the hydrogen bonding between CNCs and PBA chains. Amorphous functional groups cover the surface of CNCs. These polymer chains are locked to the surface of CNCs by hydrogen bonding, which promotes the nucleation effect and intensifies the formation of α crystal. The dispersion of the α-type ordered structure in the melt due to the “memory effect” also contributes to the thickening of tensile lamellar after the initial crystal formation. Sukyai P et al. [6] added different amounts of CNCs to the whey protein (WPI) solution and made films. The introduction of CNCs increased hydrophilicity and tensile strength and Young’s modulus of the composite film. The resistance of the film to water vapor and oxygen decreases with the increase of CNCs content, therefore, a film with 8% CNCs provided appropriate mechanical properties and barrier properties and can be utilized as a food packaging material.

Sodium alginate (SA) widely exists in the cell-matrix and cell wall of seaweed cells [7]. It is a block copolymer randomly formed by the linear connection of β-D-mannuronic acid and α-L-guluronic acid [8]. Each structural unit contains O_2(n)_H and O_3(n)_H, which provides multi-point interaction of intermolecular and intramolecular hydrogen bonds. SA is widely used in dye adsorption [9], wound dressing [10], drug sustained-release [11], nerve scaffold [12], etc. Antarctic krill protein (AKP) contains nitrogen atoms, benzene rings, and ether bonds, forming hydrogen bonds with hydroxyl groups in SA. Therefore, mixing SA and AKP can produce complex interactions and improve the performance of a single seaweed fiber to a certain extent. Yang et al. [13]. Prepared SA/AKP composite fiber by blending method, found that the strength of intermolecular hydrogen bonds of SA/AKP fiber increased with the increase of AKP content, and the percentage of intermolecular hydrogen bonds decreased with pH increasing from 6 to 9. The crystallization of composite material increased as intramolecular hydrogen bonds increased. However, due to loose cross-linking of the alginate system and the absorption of AKP crystal structure by composite system [14], SA/AKP composite fiber has low crystallinity and poor water resistance and mechanical properties.

In this paper, CNCs with a high aspect ratio was added into the SA/AKP composite system for the first time, aiming to use the hydroxyl group of CNCs to form dynamic hydrogen bonds with SA and AKP, and cross-link with the ions of SA and AKP to form multiple cross-linked structures, to improve the mechanical properties and water resistance of composite fiber. Furthermore, to improve the research efficiency, the relationship between the structural viscosity index and spinnability in rheology was used to select the spinning conditions, explored the influence of temperature and CNCs concentration on the structure and properties of composite fiber under the guidance of the structural viscosity index, designed to improve the mechanical properties and water-resistance of SA/AKP/CNCs composite fiber, the product is expected to be applied in biomedical and biomimetic materials.

## 2. Results and Discussion

### 2.1. Rheological Properties of SA/AKP/CNCs Composite Solution

**Figure 1:**
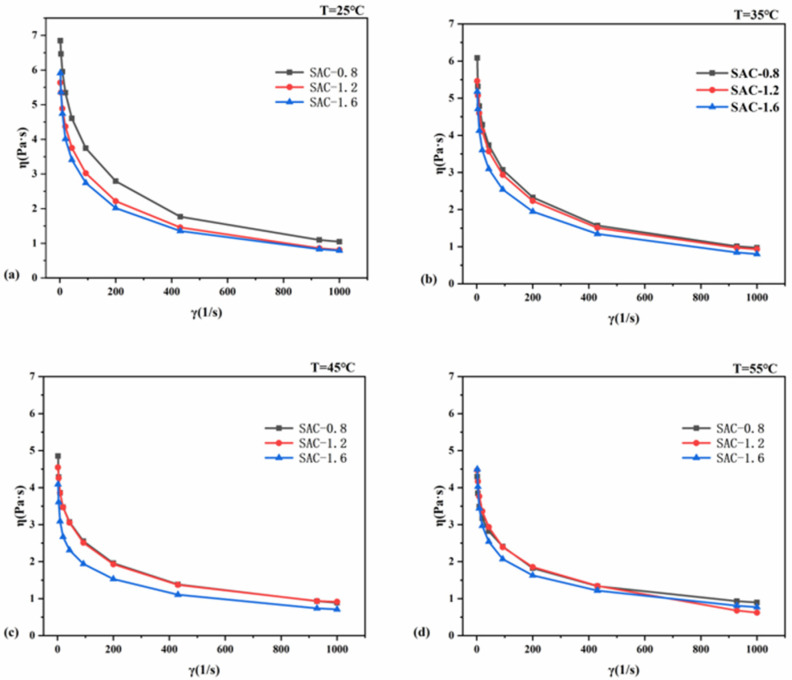
Relationship between η and γ of SA/AKP/CNCs composite solution at different spinning temperature (**a**) T = 25 °C, (**b**) T = 35 °C, (**c**) T = 45 °C, (**d**) T = 55 °C.

As shown in Figure 1a–d, the apparent viscosity of SAC-0.8, SAC-1.2, and SAC-1.6 has a slightly different sensitivity to shear rate, but all of them decrease with the increase of shear rate. The three groups of solutions all belong to non-Newtonian fluid with shear-thinning. The theory of entanglement can explain the reasons for the above phenomenon. The mixed solution is a transient network system. The concentration of entanglement point after the system reaches dynamic equilibrium is related to the given conditions (such as shear stress, shear rate, etc.). with the increase of shear rate, the entanglement between molecular chains opens, the concentration of entanglement point decreases, the flow resistance decreases, and the apparent viscosity also decreases. In addition, when the shear rate increases, the desolvation of macromolecules may occur, the contact area of macromolecular chains decreases, and the apparent viscosity decreases [15]. The apparent viscosity of the three groups of samples decreases with the increase of temperature, which is because the internal energy of molecules increases with the increase of temperature, and the constraint of molecular interaction force is not enough to limit the stronger and stronger molecular movement, the intermolecular distance increases, the molecular attraction decreases, the intermolecular friction decreases, and the apparent viscosity decreases. The apparent viscosity of the three groups of samples decreases with the increase of CNCs content, which is due to the rigidity of CNCs molecular chain, CNCs rigid molecular chains are inserted into the macromolecular network of composite fibers, limiting the movement of molecular chains, reducing intermolecular friction and apparent viscosity.

The curves of shear stress and shear rate (lgτ-lgγ) of three groups of spinning stock solution were made of different temperatures, the slope was non-Newtonian index (Table 1) and the formula was as follow.

$$\tau =K{\dot{\gamma }}_{}^{n}$$

where *K* is the consistency coefficient, *n* is a non-Newtonian index.

As shown in Table 1, the non-Newtonian index of three fluids was all less than 1, indicating that they belonged to pseudoplastic fluid. The non-Newtonian index increased with the increase of temperature generally, which is because, with the temperature increases, the macromolecular chain absorbs energy, the movement of the macromolecular chain motion unit increases, the relaxation effect of the fluid is enhanced due to the sheer force, the relaxation time is reduced, and the shear orientation effect is weakened, so the non-Newtonian property is weakened and the non-Newtonian index increases [16]. The non-Newtonian index decreases with the increase of CNCs content, which is because CNCs form more entangled networks with SA and AKP, so the non-Newtonian property is enhanced.

**Figure 2:**
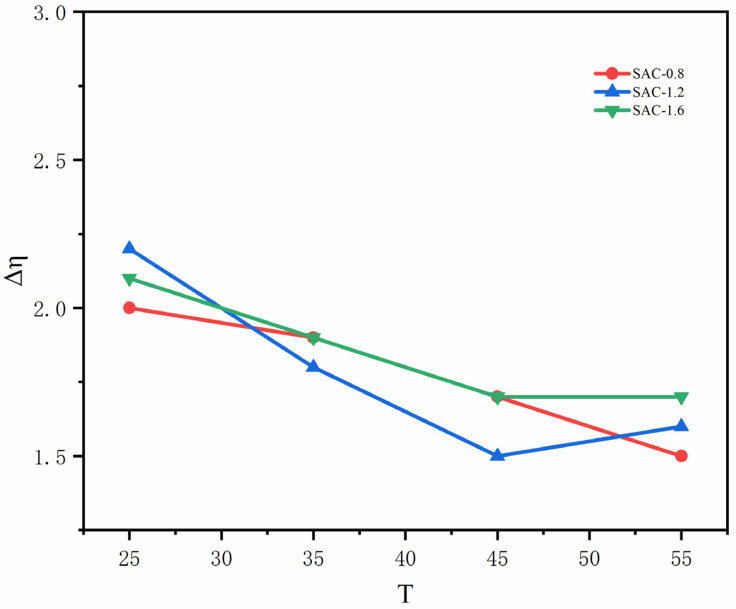
The structural viscosity index of SA/AKP/CNCs composite solution.

The structural viscosity index can represent the structurization degree of spinning fluid, and it is a crucial scale to measure the spinnability of spinning fluid [15,16]. The smaller Δη is, the smaller the structurization degree of the spinning stock solution is, and the better spinnability is. Figure 2 shows the structural viscosity index of three groups of samples at different temperatures, the Δη of SAC-0.8 at 55 °C and the Δη of SAC-1.2 at 45 °C is 1.5, compared with other spinning temperatures, the spinnability of these two groups is the best. To further clarify the influence of temperature and CNCs concentration under the optimum spinnability on the spun fiber, SAC-0.8 and SAC-1.2 were spun into the fiber at 45 °C and 55 °C for further testing.

### 2.2. Infrared Spectrum Analysis of SA/AKP/CNCs Composite Fiber

**Figure 3:**
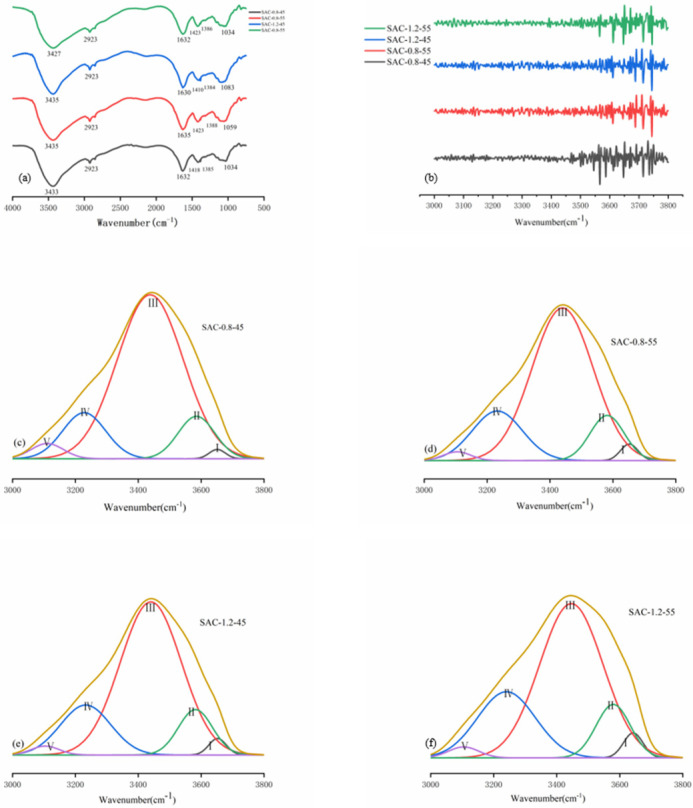
(**a**) The infrared spectrum of SA/AKP/CNCs composite fiber, (**b**) the second derivative spectrum of SA/AKP/CNCs composite fiber, Gauss curve fitting of SA/AKP/CNCs composite fiber by different types of hydrogen bonds (**c**) SAC-0.8-45, (**d**) SAC-0.8-55, (**e**) SAC-1.2-45, (**f**) SAC-1.2-55.

Figure 3a shows the Fourier transform spectrum of four groups of SAC-0.8-45, SAC-0.8-55, SAC-1.2-45, and SAC-1.2-55. As shown in Figure 3a, the characteristic peak of the four groups was the same. Among them, the peak at 1034–1083 cm^−1^ represented the ν(C-C) stretching vibration, the absorption peak at 2923 cm^−1^ was attributed to ν(C-H) stretching vibration, the broadband at 3425–3427 cm^−1^ mainly was ν(O-H) stretching vibration, the peak at 1632 cm^−1^, 1423 cm^−1^ and 1386 cm^−1^ represented, respectively, the vibration ν(C=O), δ(N-H) and ν(C-N) namely amides I, II, and III [17]. By comparison, it was found that although the infrared absorption peak of the hydroxyl group and the protein-amide group were shifted with different groups, no new absorption peak appeared, so the reason for the shift is the hydrogen bonding between the hydroxyl group and protein. With the change of CNCs concentration and extrusion temperature, the intramolecular and intermolecular hydrogen bonds will be affected accordingly.

**Figure 4:**
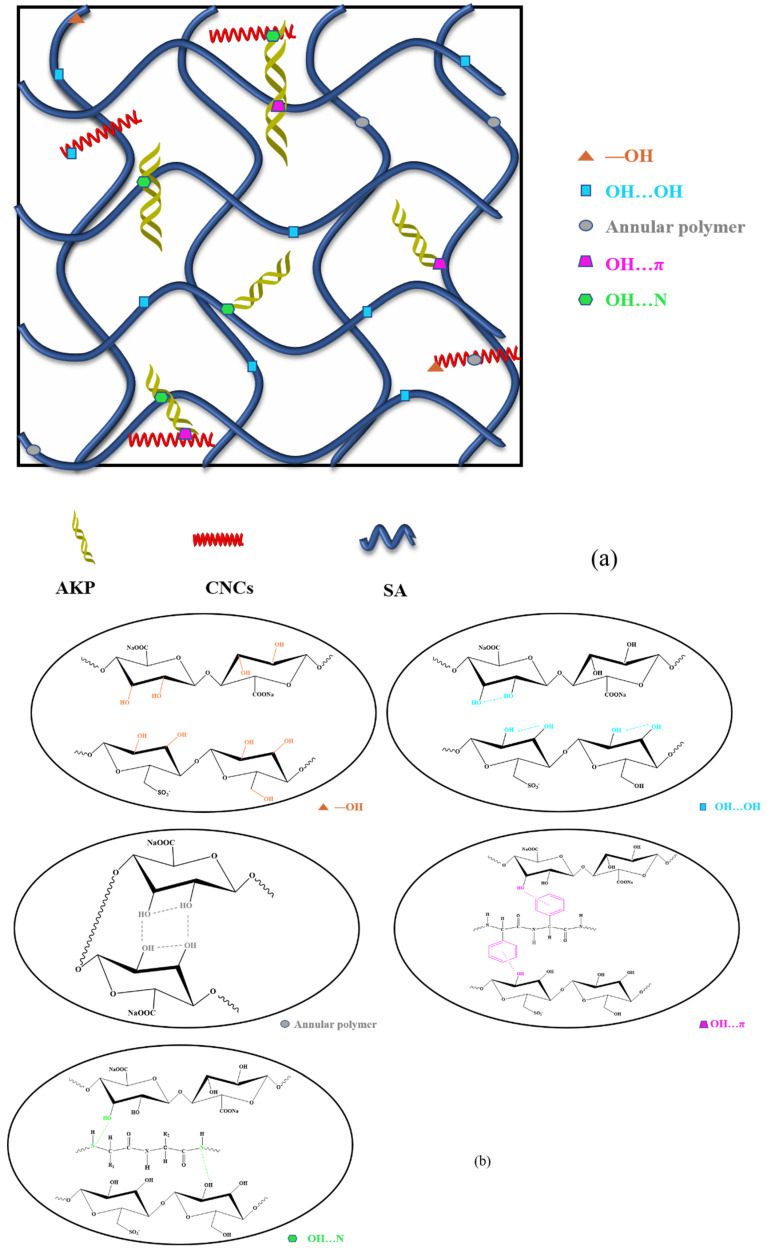

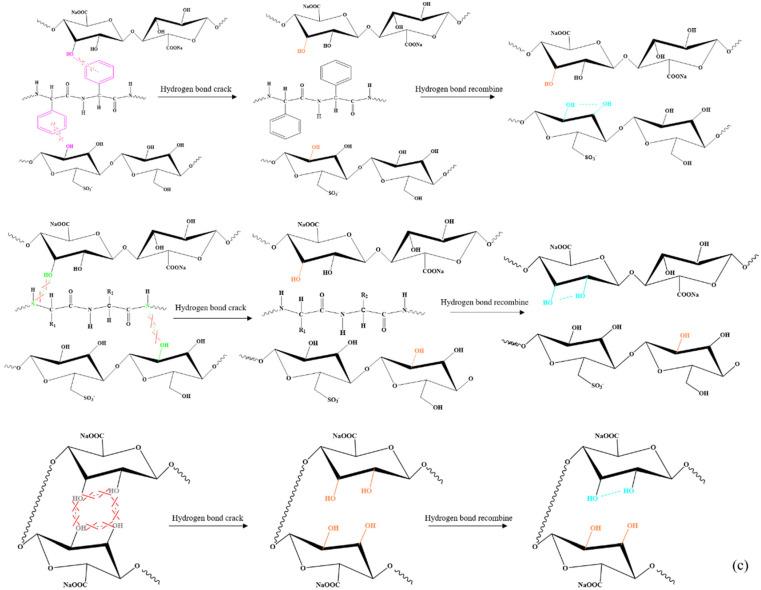
(**a**) The dynamic hydrogen bonds of SA/AKP/CNCs composite fiber, (**b**) different types of hydrogen bonds, (**c**) Fracture and recombination of hydrogen bonds.

To further clarify the relationship between them, we performed infrared peak fitting for the broadband of hydroxyl at 3000–3800 cm^−1^. Different hydrogen bond types were determined by the second derivative spectrum in Figure 3b. The distribution and strength of different types of hydrogen bonds were all subject to Gaussian fitting [13]. Relevant results were shown in Figure 3c–f and Table 2, Table 2 was a summary of the data in Figure 3c–f. The percentages of hydrogen bonds are determined by the percentage of the area of each curve in the figure, as shown in the last column of Table 2. A dynamic hydrogen bond network is shown in Figure 4a, and different types of hydrogen bonds were shown in Figure 4b. By comparing the four groups of fiber, it could be seen intuitively that the change of spinning temperature would affect the hydrogen bonds of the fiber with the same CNCs content. When the temperature rose from 45 °C to 55 °C, from SAC-0.8-45 to SAC-0.8-55, the intermolecular hydrogen bond content decreased from 13 to 12% the intramolecular hydrogen bond content increased from 85.5 to 86% and the free hydroxyl content increased from 1.5 to 2%. From SAC-1.2-45 to SAC-1.2-55, the intermolecular hydrogen bond content decreased from 14 to 13%, the intramolecular hydrogen bond content increased from 84 to 85%, and the free hydroxyl content stayed the same. The reason for the above changes is that the intermolecular hydrogen bond between SA, AKP, and CNCs is weakened with the increase of temperature (OH…π and OH…N exist only between hydroxyl and AKP). With the increase of temperature, the macromolecule absorbs energy, the thermal motion intensifies, and the intermolecular distance increases, finally, resulting in the fracture of OH…π and OH…N, as shown in Figure 4c. The intermolecular hydrogen bond breaks to form the free hydroxyl, and the free hydroxyl is not enough to recombine with another molecule due to the stretch of the molecular chain. So, some of the free hydroxyls are converted to intramolecular hydrogen bond OH…OH. Due to the intramolecular hydrogen bond, Annular polymer is very unstable. The free hydroxyl will not form this type of hydrogen bond. At the same time, this kind of hydrogen bond will also break down to form the free hydroxyl with the increase of molecular thermal motion. In conclusion, the increase of temperature leads to the reduction of intermolecular hydrogen bonds, which are partially converted to intramolecular hydrogen bonds, and partially exist in the form of free hydroxyl.

By comparing SAC-0.8-45 and SAC-1.2-45, SAC-0.8-55 and SAC-1.2-55, we found that different content of CNCs would also affect the hydrogen bonds at the same spinning temperature. As the content of CNCs increases, the content of intermolecular hydrogen bonds increases, and the connection between CNCs, SA, and AKP becomes closer. Meanwhile, due to the intermolecular hydrogen bond, the molecular chain of fiber stretches, and the rigidity of the molecular chain is also improved. 

### 2.3. X-ray Diffraction Analysis of SA/AKP/CNCs Composite Fiber

**Figure 5:**
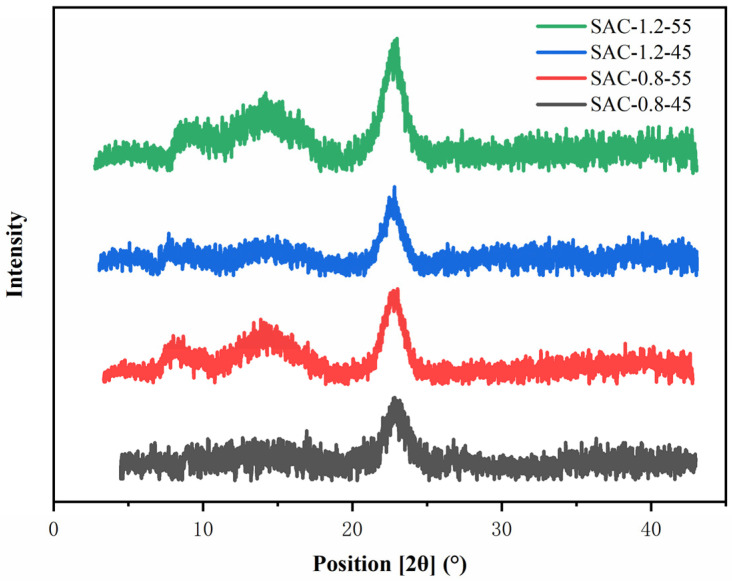
The XRD spectrum of SA/AKP/CNCs composite fiber.

The XRD pattern of SA/AKP/CNCs composite fiber was shown in Figure 5, Figure 5 showed that 2θ = 8.32° was the lattice diffraction peak of AKP [18], 2θ = 14.21° and 2θ = 22.52° were lattice diffraction peaks of cellulose [19], which were different from SA/AKP composite fiber [13]. SAC-0.8-45, SAC-0.8-55, SAC-1.2-45 and SAC-1.2-55 all showed sharp and robust diffraction peaks at 2θ = 22.52°. However, SAC-0.8-45 and SAC-1.2-45 had no firm diffraction peaks at 2θ = 8.32° and 2θ = 14.21°, while SAC-0.8-55 and SAC-1.2-55 had relatively strong diffraction peaks at 2θ = 8.32° and 2θ = 14.21°. The crystallinity of four samples is shown in Table 3, by fitting the crystallization peak and the amorphous peak, we calculated the crystallinity by JADE 6. The crystallinity of the SA/AKP/CNCs composite fiber increased with the increase of CNCs content and spinning temperature, the maximum crystallinity of the SA/AKP/CNCs composite fiber is 27%, the crystallinity increases by 42.1–92.2%. The above phenomenon can be explained by the blending compatibility theory of polymer materials, most polymer blends have a lower critical miscibility temperature. The system is completely compatible when it’s lower than this temperature, and partially compatible when higher than this temperature [20]. Crystallization is divided into two stages: one is the formation of the crystal nucleus, the other is crystal growth. CNCs exist in the form of nanocrystals in the system, in spinning and forming, CNCs can be used as nucleation sites, and the more sites, the more conducive to crystallization. The crystallinity increases with the increase of temperature mainly because with the increase of temperature, the movement ability of macromolecular chains is stronger, and it is easier to make directional arrangements on the surface of CNCs. What is more, the higher spinning temperature increases the fluidity of the filament, as shown in Figure S1. At the same time, the transition from orientation to crystal order requires lower energy and is more conducive to the improvement of crystallinity [21].

The change of fiber crystallinity is also consistent with the change of intermolecular hydrogen bonds. The intermolecular hydrogen bond strengthens the connection between molecular chains and forms more entanglements, which is not conducive to the formation of crystallization. However, the intramolecular hydrogen bond improves the regularity of molecular chains, makes molecular chains more easily arranged on the lattice, and provides more nucleation sites for the growth of the crystal, which is conducive to the formation of crystal.

As shown in Figure S1, the diffraction arc of the four groups of composite fiber is brighter at the equatorial line, and the dispersion point points along the equatorial line at a small angle can be seen. Studies have shown that the strength of the small angle comes from the tensile lamellar composed of the crystalline phase and the amorphous phase of the semi-crystalline polymer [22], so it can be proved that the molecular chain of the composite fiber is oriented along the fiber axis (tensile direction) and has a one-dimensional periodicity.

### 2.4. Mechanical Properties Analysis of SA/AKP/CNCs Composite Fiber

**Figure 6:**
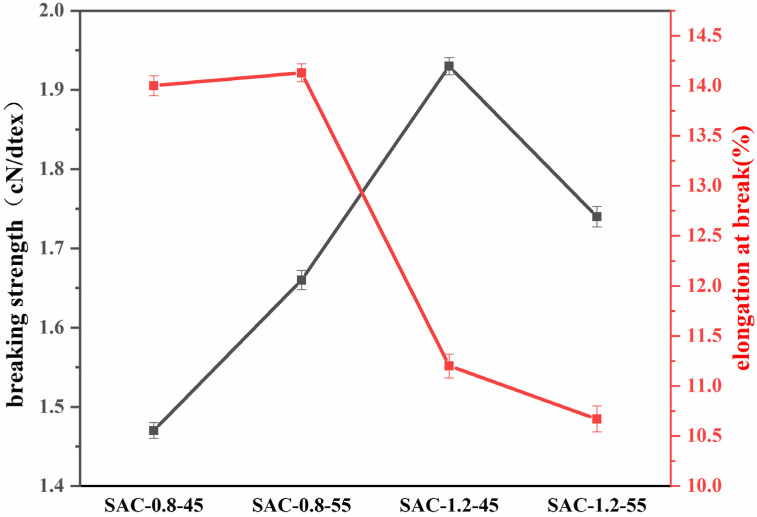
The tensile strength and elongation of SA/AKP/CNCs composite fiber.

The mechanical properties of SA/AKP/CNCs composite fiber were shown in Figure 6, Figure 6 showed that when the CNCs content was 1.2% and the spinning temperature was 45 °C, SAC-1.2-45 had the maximum strength and the breaking strength was 1.93 cN/dtex, indicating that the fiber prepared under this spinning condition has a dense structure and requires a strong force to break its chemical bonds.

By comparing SAC-0.8-45 and SAC-1.2-45, SAC-0.8-55 and SAC-1.2-55, it could be seen that the content of CNCs increased from 0.8% to 1.2%, the breaking strength and elastic modulus of composite fiber were significantly improved, the breaking strength increased by 31%, the addition of CNCs played an essential role in enhancing the composite fiber, which is because CNCs has a high aspect ratio and contains a large number of free hydroxyl, which can form a large number of hydrogen bonds with the connection between molecular chains, the Van der Waal’ force (VDW) needed to overcome the fracture of composite fiber is increased, so the mechanical properties of the fiber are improved. In addition, CNCs have high crystallinity, and the tensile lamellar formed by CNCs and the amorphous of the composite fiber improve the crystallinity of the composite fiber, which improves the mechanical properties of the composite fiber. The mechanical properties of the composite fiber are improved, and the corresponding elongation at break is decreased, which is consistent with the theory [23].

**Figure 7:**
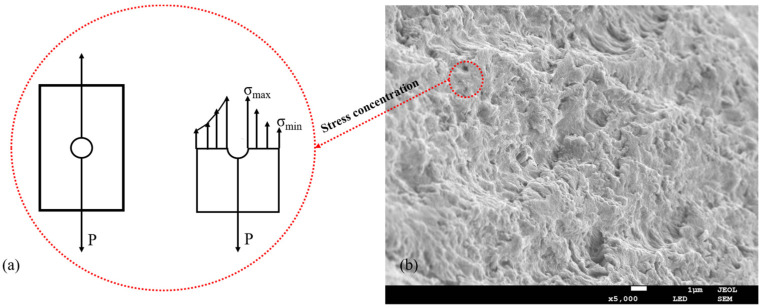
(**a**) The schematic presentation of the stress concentration of SA/AKP/CNCs composite fiber, (**b**) the cross-section morphology of SA/AKP/CNCs composite fiber.

Compared with SAC-0.8-45 and SAC-0.8-55, when the spinning temperature rose from 45 °C to 55 °C, as mentioned above, the crystallinity of composite fiber increased, the breaking strength and elastic modulus of composite fiber were improved. However, we found that in SAC-1.2-55, compared with SAC-1.2-45, the crystallinity was improved, but the mechanical properties were decreased. The cause of the above phenomenon is stress concentration. The mechanical properties of the fiber prepared by wet spinning are regulated by aggregation structure and morphology structure. When the temperature rises to 55 °C, molecules acquire more energy, and their motion intensifies, the intense double diffusion during fiber molding results in uneven morphology of fiber interior and surface, with more microporous structures, namely defects, as shown in Figure 7b. The existence of these microporous defects will lead to uneven distribution of the material under stress, and the stress near the defects will increase sharply, leading to fiber fracture, as shown in Figure 7a. Thus, it can be seen that for wet spinning, the influence of macroscopic morphological structure on the mechanical properties of fiber is more significant than that of supramolecular structure. Therefore, attention should be paid to the control of the spinning process and then realized the control of fiber macroscopic morphological structure.

### 2.5. Water Resistance Analysis of SA/AKP/CNCs Composite Fiber

**Figure 8:**
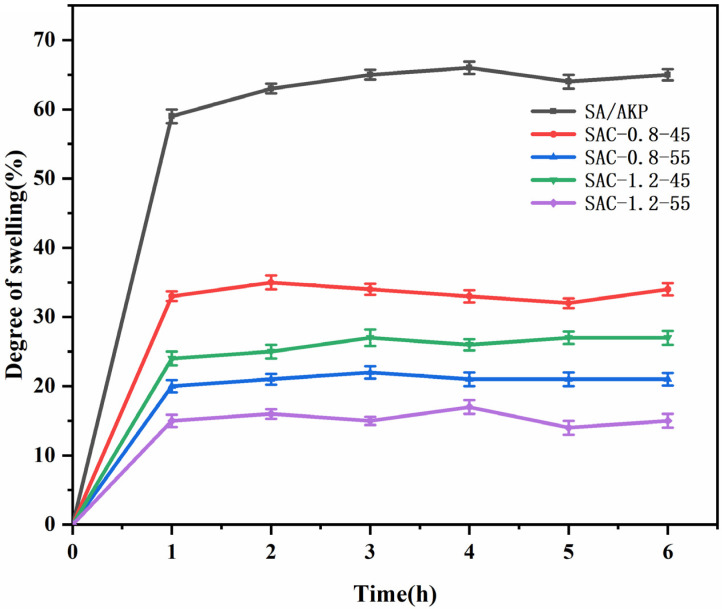
Water absorption of SA/AKP composite fiber and SA/AKP/CNCs composite fiber.

The water-resistance of SA/AKP composite fiber and SA/AKP/CNCs composite fiber was shown in Figure 8, the swelling degree of the two composite fibers was almost unchanged after one h, indicating that they had reached hygroscopic equilibrium. However, the water-resistance of SA/AKP/CNCs composite fiber was significantly improved compared with SA/AKP composite fiber, and the swelling degree of SA/AKP/CNCs composite fiber was only 21–51% of SA/AKP composite fiber, the minimum swelling rate of SA/AKP/CNCs composite fiber is 14%, indicating that the introduction of CNCs successfully improves the water-resistance of composite fiber. The reasons for the above phenomenon can be explained from two aspects: The first is to improve the crystal structure of SA/AKP/CNCs composite fiber. The adsorption of water is in the amorphous region of the fiber. Therefore, the increase of crystallinity and one-dimensional orientation along the fiber axis make the structure of the composite fiber compact, which reduces the accessibility of the composite fiber to the water molecule and inhibits the swelling of the composite fiber. The other is due to the hydrogen bonding between CNCs and SA. As mentioned above, SA/AKP/CNCs composite fiber has a large number of intramolecular and intermolecular hydrogen bonds formed by hydroxyl, and the content of the remaining unreacted free hydroxyl is only 1.6–3.7%, so only a small number of free hydroxyls can be used as hydrophile groups to bond with water molecules. In conclusion, the two aspects work together to improve the water resistance of SA/AKP/CNCs composite fiber. At the same time, we compared SAC-0.8-45, SAC-0.8-55, SAC-1.2-45, and SAC-1.2-55. As a result, we found that the crystallinity and water resistance followed the same rule: the higher the crystallinity, the better the water resistance, which was confirmed with our theory.

### 2.6. Scanning Electron Microscopy Analysis of SA/AKP/CNCs Composite Fiber

**Figure 9:**
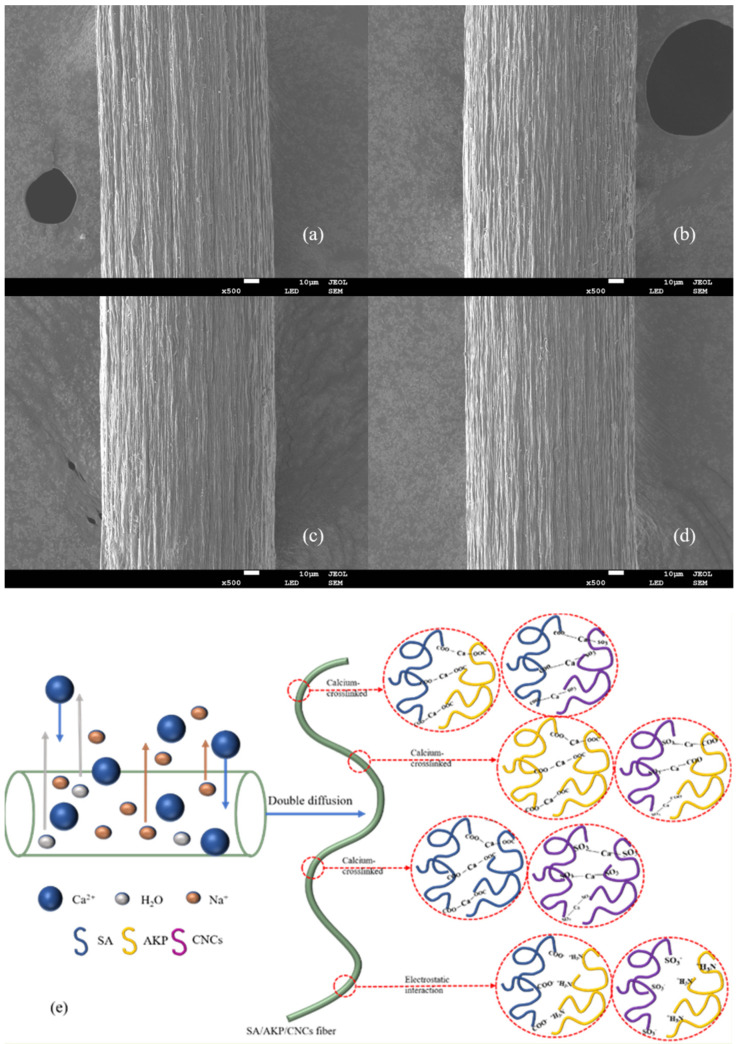
The surface morphology of SA/AKP/CNCs composite fiber, (**a**) SAC-0.8-45, (**b**) SAC-0.8-55, (**c**) SAC-1.2-45, (**d**) SAC-1.2-55, (**e**) double diffusion and ionic crosslinking of SA/AKP/CNCs composite fiber.

The surface morphology of composite fiber was shown in Figure 9a–d. There were 1–2 μm furrow structures on the surface of the four groups of fibers, which were caused by the double diffusion between the fiber and the coagulation bath during the wet spinning. When the composite fiber is extruded into the coagulation bath by spinneret, SA, AKP and CNCs components on the surface of the composite fiber react with Ca^2+^ rapidly, since Ca^2+^ is 2-valent, there are various cases of Ca^2+^ cross-linking with SA, AKP, and CNCs, simultaneously or to SA, AKP and CNCs alone, in addition, due to polyelectrolyte effect, SA, AKP, and CNCs also have electrostatic adsorption, the above conditions act simultaneously to form ionic crosslinking structures, as shown in Figure 9e. However, the fiber core layer was still in an incomplete cross-linking state because Ca^2+^ was not wholly entered. When Ca^2+^ in the coagulation bath entered the fiber, the water inside the fiber also penetrated the surface and entered the coagulation bath; at this time, because of double diffusion inside and outside the fiber, negative pressure is generated inside the fiber, so the surface of composite fiber shrinks and gully structure is formed [24].

## 3. Conclusions

In summary, CNCs are introduced into SA/AKP system as reinforcement material. Under the guidance of the structural viscosity index in rheological theory, SA/AKP/CNCs composite fiber with improved mechanical properties, crystallinity, and water resistance has been successfully prepared by adjusting spinning temperature and CNCs concentration. It is found that when the concentration of CNCs increases from 0.8% to 1.2%, the intermolecular hydrogen bond content of SA/AKP/CNCs composite fiber increases, the maximum value is 36.7%, the crystallinity of SA/AKP/CNCs composite fiber increases significantly, the maximum value is 27%, the breaking strength of SA/AKP/CNCs composite fiber increases, and the maximum value is 1.93 cN/dtex. When the spinning temperature increase from 45 °C to 55 °C, the intramolecular hydrogen bond content of SA/AKP/CNCs composite fiber increases by 62.2%, the crystallinity increases by 42.1–92.2%, the water-resistance of SA/AKP/CNCs composite fiber improves obviously, the minimum swelling degree is 14%, which is only 21.5% of SA/AKP composite fiber.

## 4. Materials and Methods

### 4.1. Materials

CNCs (Prepared by sulfuric acid hydrolysis with sulfonic acid groups on the surface, Dh was 92 nm, length was 200 nm, the diameter was 19 nm) were supplied by Shanghai ScienceK Co., Ltd., Shanghai, China. SA with a molecular weight of 3 × 10^6^–5 × 10^6^ was obtained from Qingdao Bright Moon Seaweed Group Co., Ltd., Qingdao, China. AKP (particle size was 200 μm to 1000 μm) was extracted from the alkali solution of Antarctic krill, then filtrated and dried [25]. Sodium hydroxide (NaOH) and hydrochloric (HCl) were analytical grade, from Tianjin Ruimingte Chemicals co., Ltd., Beijing, China.

### 4.2. Preparation of SA/AKP/CNCs Spinning Solution

First of all, added 0.8 g, 1.2 g, and 1.6 g CNCs to 100 g water, respectively, and emulsified for 20 min by a high-speed dispersion homogenizer to evenly disperse the CNCs solution. Then, the AKP/CNCs composite solution was prepared by adding 1 g AKP and 0.5 g NaOH into CNCs solution, heating and stirring in a 60 °C water bath for 60 min. Finally, the SA/AKP/CNCs composite solution was obtained by adding 4 g SA into AKP/CNCs composite solution, stirring for 120 min, adjusting pH to 7 with HCl. The ratio of SA to AKP was 4:1, the amount of CNCs was 0.8, 1.2, and 1.6% of the AKP, which were recorded as SAC-0.8, SAC-1.2, and SAC-1.6, respectively.

### 4.3. Preparation of SA/AKP/CNCs Composite Fiber

Under the guidance of the rheological theory of composite materials, SAC-0.8 and SAC-1.2 with low structural viscosity index were optimized. Then, by using self-made wet spinning equipment, two groups of samples were squeezed into 5% CaCl_2_ at 45 °C and 55 °C, respectively, stayed for 3 min, washed with deionized water to remove the surface CaCl_2,_ and stretched 1.8 times in a water bath at 90 °C and dried for 24 h, finally, the composite fiber was obtained, marked as SAC-0.8-55, SAC-1.2-45 and SAC-1.2-55, respectively.

### 4.4. Rheological Property Test

The rheological properties of SA/AKP/CNCs solution were studied by a digital rotational viscometer (DV-C, BROOKFIELD). Test condition: 25–55 °C, 12-100 RPM. The structural viscosity index (Δη) was calculated by formula.

$$\Delta \eta =\left(-\frac{dlg\eta_{\alpha }}{d{\dot{\gamma }}_{c}^{1/2}}\right)$$

where Δη is the structural viscosity index, 1; $\eta_{\alpha }$ is the apparent viscosity, Pa s; ${\dot{\gamma }}_{c}$ is true sheer rate, 1/s.

### 4.5. Fourier Transform Infrared Analysis

The Fourier transform infrared spectroscopy of the SA/AKP/CNCs composite fiber was obtained with Fourier transform infrared spectrometer (Thermo Scientific Nicolet iS50, Waltham, MA, USA). The sample was prepared in the usual way of about 1 mg of the sample mixing with 100 mg of KBr.

### 4.6. X-ray Diffraction Analysis

The crystal structure of composite fiber was measured by A D8 Advance X-ray diffractometer (Bruker AXS). The intensity and current of the generator were 40 kV and 200 mA. The fiber sample was scanned from 5 to 45° at a rate of 5°/min.

### 4.7. Tensile Test

Tensile properties of the SA/AKP/CNCs composite fiber were measured using an electronic single fiber strength meter (LLY-06ED). Taking 50 mm length of the fiber. The test was performed at 20 °C, 65% R.H. The date quoted was the average of 10 tests.

### 4.8. Water Absorption Test

The 50 mm SA/AKP/CNCs composite fiber sample was cut and dried in an oven at 60 °C for 24 h, the weight *W*_0_ was taken out, and then the sample was soaked in deionized water, the weight *W*_1_ was taken out every 1 h, and the water absorption (*K*) could be obtained from the formula. The date quoted was the average of 10 tests.

$$K=\frac{W_{1}-W_{0}}{W_{0}}$$

where *K* is the water absorption, %; $W_{0}$ is the weight of the sample before soaking, g; $W_{1}$ is the weight of the sample after soaking, g.

### 4.9. Scanning Electron Microscopic Analysis

The morphology of the SA/AKP/CNCs composite fiber was captured using a scanning electron microscope (S-4800, HITACHI) at the accelerating voltage of 10 kV after sputter-coating with Au.

## Figures and Tables

**Table 1:** The non-newtonian index of SA/AKP/CNCs composite solution.

Sample	25 °C	35 °C	45 °C	55 °C
SAC-0.8	0.69	0.71	0.73	0.75
SAC-1.2	0.68	0.71	0.74	0.73
SAC-1.6	0.67	0.70	0.72	0.72

**Table 2:** The fitting results of various kinds of hydrogen bonds.

Sample		Hydrogen Bond Type	Abbreviations	Wave Number/cm^−1^	Relative Strength/%	
SAC-0.8-45	I	Free hydroxyl	-OH	3651	1.5	1.5
	III	Intramolecular hydrogen bond	OH…OH	3438	72	85.5
	IV		Annular polymer	3227	13.5	
	II	Intermolecular hydrogen bond	OH…π	3585	10	13
	V		OH…N	3110	3	
SAC-0.8-55	I	Free hydroxyl	-OH	3652	2	2
	III	Intramolecular hydrogen bond	OH…OH	3439	69	86
	IV		Annular polymer	3232	17	
	II	Intermolecular hydrogen bond	OH…π	3581	11	12
	V		OH…N	3104	1	
SAC-1.2-45	I	Free hydroxyl	-OH	3652	2	2
	III	Intramolecular hydrogen bond	OH…OH	3440	67	84
	IV		Annular polymer	3232	17	
	II	Intermolecular hydrogen bond	OH…π	3581	12	14
	V		OH…N	3104	2	
SAC-1.2-55	I	Free hydroxyl	-OH	3642	2	2
	III	Intramolecular hydrogen bond	OH…OH	3446	61	85
	IV		Annular polymer	3240	24	
	II	Intermolecular hydrogen bond	OH…π	3579	11	13
	V		OH…N	3104	2	

**Table 3:** The crystallinity of SA/AKP/CNCs composite fiber.

Sample	Crystallinity (%)
SAC-0.8-45	14
SAC-0.8-55	19
SAC-1.2-45	17
SAC-1.2-55	27

## Data Availability

The date presented in this study are available on request from the corresponding author.

## Supplementary Materials

The following supporting information can be downloaded at https://www.mdpi.com/article/10.3390/gels8030139/s1: Figure S1. The 2D WAXD patterns of SA/AKP/CNCs fiber.
