# Supplementary figures and images for: Characteristics of Sodium Alginate/Antarctic Krill Protein Composite Fiber Based on Cellulose Nanocrystals Modification: Rheology, Hydrogen Bond, Crystallization, Strength, and Water-Resistance
Source: Gels. 2022 Feb 22;8(3):139. doi: 10.3390/gels8030139 (PMC8953857; doi:10.3390/gels8030139)

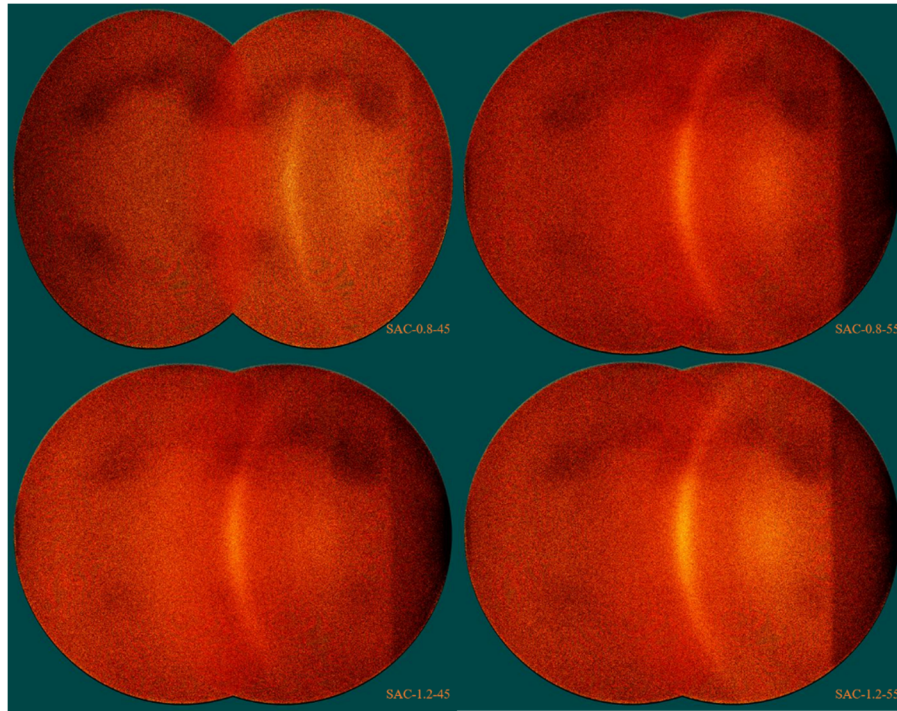

S1. the 2D WAXD patterns of SA/AKP/CNCs fiber

Supplement: Supplementary file 1 [file gels-08-00139-s001.zip › gels-1585036-supplementary.pdf]
